# A High-Continuity Genome Assembly of Chinese Flowering Cabbage (*Brassica rapa* var. *parachinensis*) Provides New Insights into Brassica Genome Structure Evolution

**DOI:** 10.3390/plants12132498

**Published:** 2023-06-29

**Authors:** Guangguang Li, Ding Jiang, Juntao Wang, Yi Liao, Ting Zhang, Hua Zhang, Xiuchun Dai, Hailong Ren, Changming Chen, Yansong Zheng

**Affiliations:** 1 Guangzhou Academy of Agricultural Sciences, Guangzhou 510335, China; liguangguang007@163.com (G.L.); huangz123@163.com (H.Z.);; 2 College of Horticulture, South China Agricultural University, Guangzhou 510642, China

**Keywords:** Chinese flowering cabbage, *Brassica rapa* var. *parachinensis*, genome structure evolution, assembly, PacBio, Hi-C

## Abstract

Chinese flowering cabbage (*Brassica rapa* var. *parachinensis*) is a popular and widely cultivated leaf vegetable crop in Asia. Here, we performed a high quality *de novo* assembly of the 384 Mb genome of 10 chromosomes of a typical cultivar of Chinese flowering cabbage with an integrated approach using PacBio, Illumina, and Hi-C technology. We modeled 47,598 protein-coding genes in this analysis and annotated 52% (205.9/384) of its genome as repetitive sequences including 17% in DNA transposons and 22% in long terminal retrotransposons (LTRs). Phylogenetic analysis reveals the genome of the Chinese flowering cabbage has a closer evolutionary relationship with the AA diploid progenitor of the allotetraploid species, *Brassica juncea*. Comparative genomic analysis of Brassica species with different subgenome types (A, B and C) reveals that the pericentromeric regions on chromosome 5 and 6 of the AA genome have been significantly expanded compared to the orthologous genomic regions in the BB and CC genomes, largely driven by LTR-retrotransposon amplification. Furthermore, we identified a large number of structural variations (SVs) within the *B. rapa* lines that could impact coding genes, suggesting the functional significance of SVs on Brassica genome evolution. Overall, our high-quality genome assembly of the Chinese flowering cabbage provides a valuable genetic resource for deciphering the genome evolution of Brassica species and it can potentially serve as the reference genome guiding the molecular breeding practice of *B. rapa* crops.

## 1. Introduction

Brassica, which belongs to the Brassicaceae family, is among the most economically important *genus*, since it contains a wide range of staple vegetables and oilseed crops. Over the course of its evolution, Brassica experienced an additional whole genome-wide triplication (WGT) event after it split with *Arabidopsis* from a common ancestor [1,2]. Thus, species in the Brassica *genus* not only display great morphological and phytochemical diversity but also karyotype diversity [2,3]. Among the most agriculturally important Brassica species, there are three diploid genome types including *Brassica rapa* (AA), *Brassica nigra* (BB) and *Brassica oleracea* (CC), and three allopolyploid species which were generated by the pair combinations of the former three diploid species, including *Brassica napus* (AACC), *Brassica juncea* (AABB) and *Brassica carinata* (BBCC). These six species and their evolutionary origination and relationship with each other are well defined in a ‘triangle of U’ model [3,4].

Due to the rapid recent advances in sequencing technology, especially next-generation sequencing (NGS), a large number of Brassica species have been sequenced, but most of the genome assemblies resulted in a low contiguity. These sequenced genomes, for example those sequenced with Illumina/Roche 454 technology, including *B. rapa* var. *pekinensis* Chiifu [5], *B. oleracea* 02-12 [6], *B. oleracea* TO1000DH [7], *B. nigra* YZ12151 [4], *B. napus* [8,9,10], and *B. juncea* [3,4] had a relatively low continuity which may impede the genomic analysis especially at the complex genomic parts such as pericentromeric and centromeric regions. Recently, the application of long-read sequencing technologies, including Oxford Nanopore Technology (ONT) and Pacific Biosciences (PACBIO), to genome assembling has greatly improved the continuity of the assembled contigs. There are more and more Brassica genomes that were reported to be sequenced with long read technology with a resulting contig N50 of up to megabase size, including *B. oleracea* cultivars HDEM, *Brassica rapa* Z1 (yellow sarson) [11], *B. oleracea* var. botrytis [12], *B. napus* [13], *Brassica rapa* [14,15,16], and pak choi [17,18]. Considerable progress has been made to improve the assembly of Brassica genomes through the use of single-molecule sequencing, optical mapping, and chromosome conformation capture technologies. And the use of long-read sequencing technologies can overcome the limitations of short-read sequencing by producing long reads of tens of kilobases (kb), which span the repetitive regions in Brassica. These studies demonstrated great success in the assembly of high continuity genome assemblies (i.e., N50 > 5 Mb) [11] with long read technology in Brassica genomes. Since the great morphological and phytochemical diversity in the Brassica species, genome information from a wide range of representative Brassica species will be helpful and needed to deeply decipher the genomic variants that may contribute to the great diversity of not only the phenotype but also the karyotype of various cultivars of the species.

The Chinese flowering cabbage (*Brassica rapa* var. *parachinensis*), locally known as Caixin, Tsai Tai, Choy Sum, bok choy, or Tsai Hsin [19,20], is an important leafy and bolting stem vegetable widely grown in Asia, particularly in China, Japan, and Korea [21]. This vegetable has high nutritional value and is rich in vitamins, minerals, secondary metabolites and dietary fiber, which can confer human health-promoting effects [20]. Unlike other *B. rapa* vegetables, Chinese flowering cabbage can bolt and flower easily without strict vernalization under low temperature. Therefore, it is very important to conduct this genome sequencing and assembly to further uncover the genomic information and molecular mechanisms involved in the formation of special morphological and phytochemical characteristics of this cultivar.

In this study, we report a high continuity (N50 = 7.2 Mb) and chromosome level genome assembly for the Chinese flowering cabbage (*Brassica rapa*). It was assembled with an integrated approach using Illumina sequencing, PacBio, and high-throughput chromosome conformation capture (Hi-C) technology. The assembly resolved a large part of the pericentromeric regions of this species. In addition, genome comparison and evolutionary analysis of this genome and other representative Brassica species were conducted. The results provide novel insights into the Brassica genome structure evolution.

## 2. Materials and Methods

### 2.1. Sample Collection

**Figure 1:**
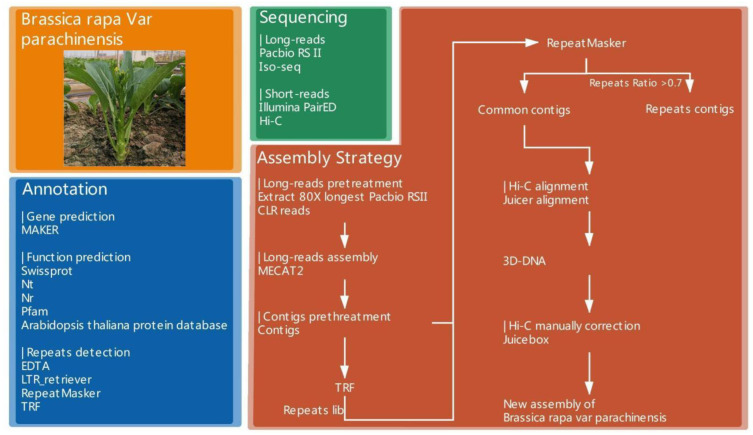
Overview of the assembly pipeline for *Brassica rapa* var. *parachinensis* genome. The steps include assembly of PacBio reads followed by scaffolding using Hi-C, and extensive QC using high coverage of Illumina short reads followed by *de novo* repeat annotation and gene annotation using ISO-seq sequencing.

Young leaves were collected from a single plant of *B. rapa* var. *parachinensis* cv. Youlv 701 (Figure 1), which is a highly inbred line issued by the Guangzhou Institute of Agriculture Science, in Guangzhou, Guangdong, China. The collected young leaves were soon frozen in liquid nitrogen and stored at −80 °C for DNA and RNA extraction.

### 2.2. DNA Extraction and Sequencing

For Illumina sequencing, the phenol/chloroform extraction protocol was used to extract DNA from 2 g of young leaves. An Illumina sequencing library for an insertion length of 250 bp was prepared using the TruSeq Nano DNA LT Library Preparation Kit (Illumina Inc., San Diego, CA, USA). DNA purity and size range were evaluated with Agilent Bioanalyzer 2100 (Agilent Technologies, Santa Clara, CA, USA). An Illumina sequencing library (PE) with an insertion length of 300–350 bp was constructed and sequenced using the Illumina HiSeq 2000 platform.

The DNA extracted from the young leaves was also used for the PacBio sequencing library construction. According to the manufacturer’s protocol (Pacific Biosciences, San Diego, CA, USA), 10 μg of Chinese flowering cabbage genomic DNA was used for a 30-kb template library preparation using the BluePippin Size Selection system (Sage Science, Waltham, MA, USA). The library was sequenced on the PacBio SEQUEL II platform.

The PacBio platform was used to generate long genomic reads for the construction of a reference genome for the Chinese flowering cabbage. After removing adaptor sequences, more than 113 Gb of subreads were obtained with a 219 times sequence coverage. The sequencing data were used for the following genome assembly operations.

### 2.3. Genome Size Estimation Based on NGS Sequencing Data

The HTQC package [22] was used to filter low-quality bases and reads. Briefly, three steps were performed to clean the NGS data. First, the adapter sequences were removed from the reads; second, the reads with more than 10% N bases were eliminated; and third, reads with more than 50% low-quality bases (<=5) were discarded. Lastly, we obtained 42.3 Gb (~86X) of cleaned data for the Kmer-based analysis. We also randomly picked 10,000 read pairs and blasted them against the NCBI non redundant nucleotide (nt) database to check for obvious sample contamination.

### 2.4. De Novo Assembly of the Chinese Flowering Cabbage Genome

The MECAT2 package [23] was used for the Chinese flowering cabbage genome assembly. Long reads had a length cutoff of 10 kb. We applied two rounds of polishing using NGS short reads with Pilon [24]. TRF (tandem repeats finder) [24,25] was used to identify the tandem repeats, and we removed the contig with higher than 60% of series repeats. The completeness of the assembled genome was evaluated using BUSCO v3.0 analysis [26].

### 2.5. Hi-C Library Preparation and Data Analysis

In the present study, 8 g of young leaf tissue collected from the same *B. rapa* var. *parachinensis* plant was used for Hi-C library construction. The Hi-C experiment consisted of the following steps: crosslinking, lysis, chromatin digestion, biotin marking, proximity ligations, cross linking reversal, and DNA purification [27]. The purified and enriched DNA was used for the sequencing library construction; the DNA was sequenced using the Illumina HiSeq 2000 platform (Illumina, San Diego, CA, USA). Assembled contigs were scaffolded using Juicer [28] and 3D-DNA [29]. MCScanX [30] was used to make a collinear comparison between scaffolds and the existing *B. rapa* genome [31]. The sequence was given a new name after exhibiting synteny to *B. rapa* z1.

We used bwa mem [32] to map two paired reads to the chromosome level genome sequence alone with these parameters “-A1-B4-E50-L0”. Then the HiCExplorer kit [33] was used to build a Hi-C contact map. Parameters for the step hicCorrectMatrix were set to “--filterThreshold-3.5 5” and the rests were kept at the default settings.

### 2.6. Single Molecule RNA Sequencing (Iso-Seq) Experiment and Data Analysis

For gene annotation of the genome, transcriptome sequencing was performed with mixed leaves and roots of a young seedling (14 days after imbibition). RNA was extracted with the TRIzol Reagent (Invitrogen, Waltham, MA, USA). The RNA quality was checked by a spectrophotometer (LabTech, Hopkinton, MA, USA) and a 2100 Bioanalyzer (Agilent Technologies, USA). The verified RNA was used for transcriptome sequencing library construction. Briefly, the mRNA was reversely transcribed using a Clontech SMARTer cDNA synthesis kit. A BluePippin Size Selection System (Pacific Biosciences of California, Menlo Park, CA, USA) was used to perform the size selection for the two libraries, sized 0–3 kb and 2–6 kb, respectively, after cDNA amplification and purification. The SMRTbell libraries were constructed according to the manufacturer’s protocol and sequenced on the PacBio SEQUEL II platform (Pacific Biosciences of California, Menlo Park, CA, USA). Last, we used SMRTLink 7.0 (https://www.pacb.com/support/software-downloads/) (accessed on 15 January 2022) to produce all the mRNA sequences for genome annotation.

### 2.7. Repetitive Element Annotation and Construction of Circos Picture

The extended de-novo TE Annotator (EDTA) [34] was used to annotate the DNATE and LTR type sequences of the genome. TRF (tandem repeats finder) [25] was used to identify the centromere sequence with 20,000 points as the threshold. Finally, the repeat sequences were annotated with MAKER [35]. MCScanX [30] was used to find the collinearity from the comparison results and generate link files. Four tracks were constructed from the outer to the inner of Circos [36], showing gene density, LTR density, DNATE density and TE density, respectively, and the collinearity within the genome was shown in the inner circle.

### 2.8. Protein Coding Gene Prediction

The Isoseq3 pipeline (https://github.com/pacificbiosciences/isoseq) (accessed on 7 February 2022) was used to process the full-length transcriptome data of the Chinese flowering cabbage to obtain the transcriptome sequence. At the same time, in order to obtain a more complete gene annotation, we integrated the annotation content of *B. juncea* [4], *B. napus* [8], *B. oleracea* [6], *B. rapa* [16] and *B. nigra* [3] as the reference gene sequence using CD-HIT-EST (https://github.com/weizhongli/cdhit) (accessed on 10 February 2022) to remove the sequence redundancy. The results of the repeats sequence found by EDTA [34] and TRF [25] were used as reference repeats to enter into MAKER [35] for five rounds of gene and repeat sequence annotation.

### 2.9. Phylogenetic Analysis

The phylogenetic relationships between the Chinese flowering cabbage and other Brassica plants were analyzed using the orthologs from single-copy genes. We used Diamond for Orthofinder to build orthogroups. 20 eudicot species’ proteomes were retrieved from the Brassica Database(brassicadb.cn). We downloaded the reference genome and gff record first. Then gffread was used for the command “gffread-g$refgenome-y$protoems $gff_record” to get all species’ proteomes. The 20 eudicot species and references are listed in the Table S1. The Orthofinder package was used to find orthogroups and single-copy genes. All the single-copy genes in one species were concatenated into a super alignment, then run through a multiple sequence alignment using the mafft program. Easyspecietree (https://github.com/Davey1220/EasySpeciesTree) (accessed on 14 February 2022) was used to generate the phylogenetic relationship between the species using the maximum likelihood method.

### 2.10. Structural Variants Analysis

Structural variations were detected using an assembly-based pipeline based on LASTZ/CHAIN/NET/NETSYNTENY tools [37,38,39,40] which is publicly available at https://github.com/yiliao1022/LASTZ_SV_pipeline. (accessed on 18 February 2022) Insertion times of the LTR-retrotransposons were estimated by the divergence time (T) between the two LTRs of each intact element with the formula: T = K/2r, where K refers to the sequence difference between the 5′-LTR and 3′-LTR of an intact LTR element and r refers to the average mutation rate. Here we used the neutral substitution rate of 1.5 × 10^−1^ per synonymous site per generation [41].

## 3. Results

### 3.1. A Highly Continuous Genome Assembly of Chinese Flowering Cabbage (B. rapa var. parachinensis)

A highly inbred line of Chinese flowering cabbage (*B. rapa* var. *parachinensis*, Figure 1) was used for the genome sequencing and assembly with deep coverage long reads and Hi-C data. The assembly pipeline for the *Brassica rapa* var. *parachinensis* genome was shown in Figure 1. DNA samples from a single plant were prepared for PacBio, Illumina, and Hi-C sequencing to avoid potential genome variability between different plants. Overall, we obtained a total of 113 Gb PacBio and 47.5 Gb Illumina raw reads (Table S2), corresponding to a 219 and 86 depth of the estimated genome size (515 Mb), respectively. A preliminary survey of the genome size, heterozygosity, GC, and transposon elements (TEs) content of this inbred line was carried out with 32 GB clean Illumina reads (Table 1; ~83 coverage) using the Kmer-based method. The genome size was estimated to be about 515 Mb with an overall GC content of 38.9% and transposon elements (TE) content of 64.1% (Table S2). Remarkably, the heterozygosity was very low, with only 0.16% that would facilitate assembly.

We applied a hybrid strategy to assemble the genome. Firstly, the MECAT2 package [23] was used for the Chinese flowering cabbage genome assembly. Secondly, long reads with a length cutoff of 10 kb were polished using NGS short reads with Pilon [24]. Finally, we obtained the final contig assembly of 384 Mb with a contig N50 length of 7.2 Mb. The genome contained 450 contigs, and the longest contig was 19.9 Mb (Table 1). The GC content for the genomic contigs were 37.6% (Table 1). The results of the coverage statistics by SAM tools suggested that the assembly of this genome is credible (Table S3). Furthermore, we found that 97.8% and 0.8% of the completed and partial genes of the total of 1440 BUSCO genes were detected in the genome, respectively, which validated the completeness of the genome (Table S4).

**Figure 2:**
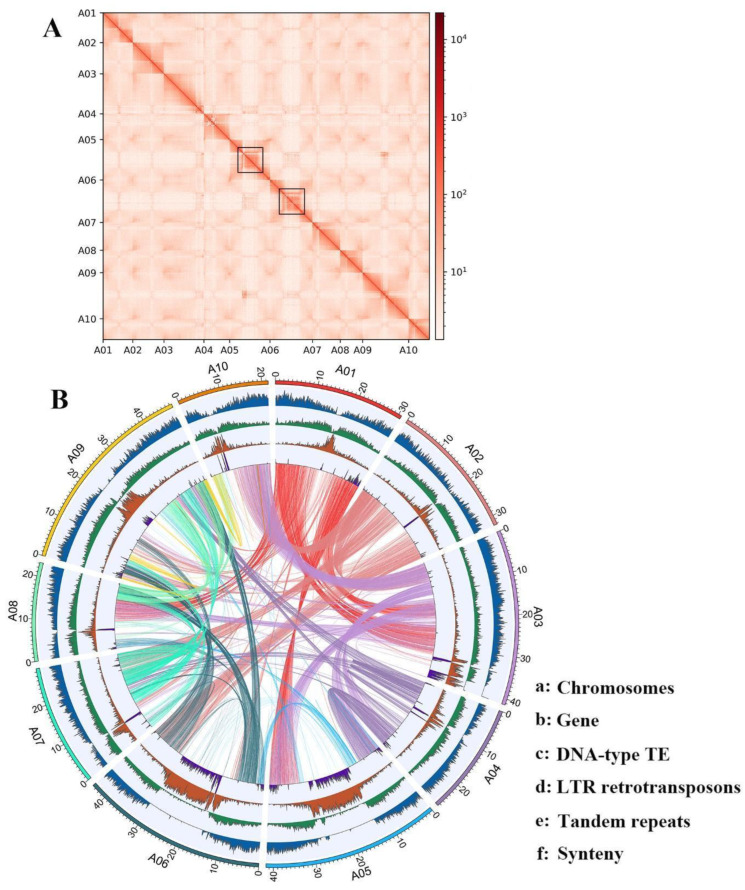
A highly continuous genome assembly of the Chinese flowering cabbage (**B**). rapa var. *parachinensis*). (**A**) Hi-C contact map of the Chinese flowering cabbage assembled chromosomes; density of Hi-C contacts are highest at the diagonals, suggesting consistency between the assembly and the Hi-C map; blue squares indicate highly repetitive pericentromeric regions on A05 and A06 chromosomes. (**B**) Circos diagram of sequence features on the chromosomes of *B. rapa* var. *parachinensis*; A01, 02, 03, 04, 05, 06, 07, 08, 09 and 10 indicate the ten assembled chromosomes of *B. rapa* var. *parachinensis*. Tracks in the circos plot from outer to inner represent a: Chromosomes; b: Gene; c: DNA-type TE; d: LTR retrotransposons; e: Tandem repeats; f: Synteny.

Furthermore, the high-throughput chromatin conformation capture (Hi-C) data was used to scaffold the contigs into a chromosome-level assembly. We obtained a total of 66 Gb cleaned Hi-C paired-end (PE) reads which is about 128 times the genome depth. Of which, 98.27% (434 M/442 M) were mappable to the current assembly and ~33.18% (147 M/442 M) were mapped to different contigs. Using the contact frequency calculated from the PE reads, 180 contigs were further folded into 10 pseudo-chromosomes (Figure 2A). These 180 contigs represent 87.93% (338 Mb/384 Mb) of the total assembled sequence and 40% (180/450) of the total contigs. The final assembly contains 69 scaffolds with a scaffold N50 of 32 Mb and the longest scaffold of 47.5 Mb in length (Table 1). The Circos map of the genome shows that each position is collinear with the other two, indicating that the annotation is complete (Figure 2B). A large number of corrected repeat regions on A05 and A06 chromosomes were identified (Figure 2B), which indicated that there might be a large region of DNA transposons and LTR transposons found at this region.

We also performed *de novo* gene prediction with guidance by homologs from the related species, using the transcriptome from short read data and full-length transcripts from ISO-seq sequencing from the present study using the MAKER pipeline [35]. We annotated 47,598 protein-coding genes in the Chinese flowering cabbage genome with an average gene length of 2060 bp (Table 1). The average number of exons per gene is 6.13, with a mean length of 199 bp (Table 1). Approximately 53.2% of the genome is annotated as repetitive sequences, which is consistent with the estimation from the Kmer-based method. LTR retrotransposons (22.26%) and DNA transposons (17.62%) are the most abundant families (Table S5).

In conclusion, we provide, to our knowledge so far, the most contiguous genome assembly of this species.

### 3.2. Gene Duplication Analysis across 20 Eudicot Genomes Reveals the Current B. rapa var. parachinensis Genome Is among the Most High-Quality Assemblies of Brassica Genomes

**Figure 3:**
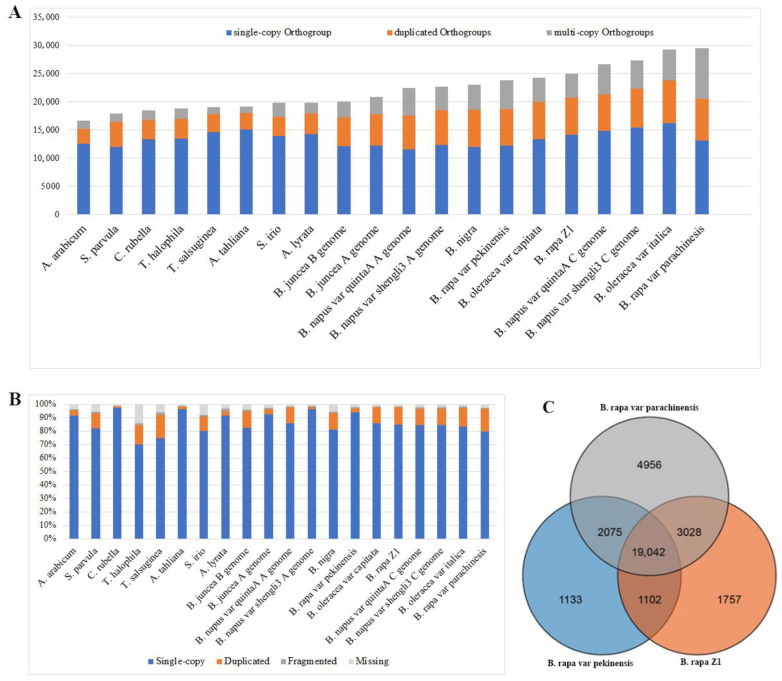
Distribution of genes in *B. rapa* var. *parachinensis* and other representative plant species. (**A**) Distribution of ortholog groups: single copy (blue), two copies (orange), and multiple copies (grey) across 20 eudicot genomes; (**B**) BUSCO analysis of genome assembly of the 20 eudicot genomes; (**C**) Venn diagram showing the overlap of gene families among Chinese flowering cabbage and two other assemblies of *B.rapa* species.

To assess the completeness of genome assembly and gene models, we used Orthofinder [42] to construct the ortholog group across 20 eudicot species and separate them into three categories: ortholog groups either with a single copy gene, two genes, or multiple (more than two) genes. The frequency of each group among the 20 eudicot species revealed that the Brassica species (i.e., *B. napus*, *B. rapa*, *B. juncea* and *B. nigra*) harbor more duplicated orthologs than *Arabidopsis* species (Figure 3A,B), which is consistent with the fact that Brassica species experienced an extra whole genome triplication (WGT) event compared with the model plant *Arabidopsis thaliana* [6]. Additionally, more duplicated orthologs are identified in the current *B. rapa* var. *parachinensis* genome assembly than in the two other assemblies of this species with a relative lower N50 (Figure 3A), suggesting that we obtained a higher N50 length of the genome assembly and a more alternative splicing annotation than previous studies [11,16]. A BUSCO analysis suggested that all the 12 Brassica species have a high quality of genome assembly and the current *B. rapa* var. *parachinensis* has the highest BUSCO value (Figure 3B).

Next, we compared the overlap of gene models among *B. rapa* var. *parachinensis* and two other *B. rapa* genomes [11,16]. A total of 19,042 genes are shared by all three genomes. The Chinese flowering cabbage genome (Figure 3C) has more specific gene models, which may be caused by the difference of assembly quality among these three genomes or their specific gene amplification history.

### 3.3. Phylogenetic Analysis of a Collection of Brassica Genomes Reveals That the Chinese Flowering Cabbage Has a Closer Evolutionary Relationship with the Diploid Progenitor of the Allotetraploid Species, B. Juncea

**Figure 4:**
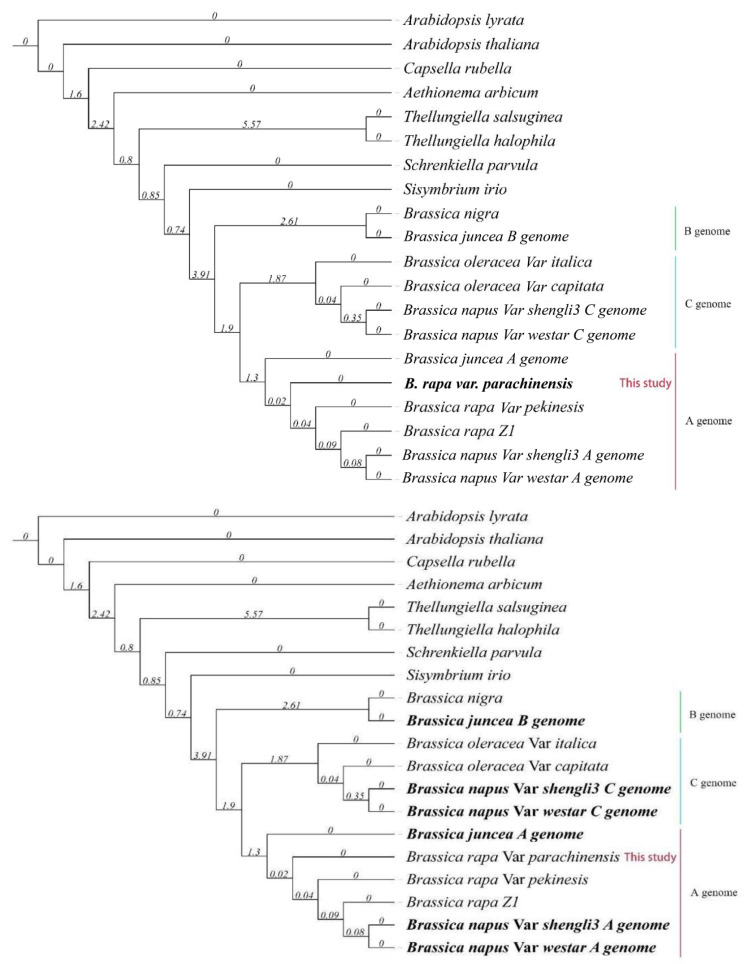
The phylogenetic relationship of *B. rapa* var. *parachinensis* with other Brassicaceae plants.

The Brassicaceae family serves as a useful model for studying polyploidy and chromosome evolution. The evolutionary relationship of six ecologically important Brassica species, including three diploid species (*B. rapa*, *B. oleracea*, and *B. nigra*) and three allotetraploid species (*B. napus*, *B. juncea*, and *B. carinata*), was well described in a classical U triangle model [2]. To elucidate the evolutionary distance of the current Chinese flowering cabbage genome to other Brassica genomes, we constructed a phylogenetic tree (Figure 4) for 12 collected Brassica genomes and eight related Brassicaceae species using the coding sequences of 434 single-copy genes that are present in all the species. The result shows that the three Brassica genome types are clearly separated from each other among the investigated species. The current Chinese flowering cabbage has an AA genome type which is closer to the AA genome of the allotetraploid species, *B. juncea*, than the AA genome of another *B. rapa* line, *B. rapa* var *pekinensis* in the phylogenetic tree, suggesting Chinese flowering cabbage is evolutionarily closer to the diploid progenitor of the allotetraploid species, *Brassica juncea*. Also, in the CC genome clade, *B. oleracea* var *capitata* was primarily the sister to two *B. napus* CC genomes and then with *B. oleracea* var *italica*, implying *B. oleracea* var *capitata* has a CC genome that is closer to the donor of CC genome of the *B. napus*. Similarly, *B. rapa* Z1 was sister first to the *B. napus* AA genome and then other AA genomes, pointing to it as being evolutionarily closer to the AA genome progenitor of *B. napus*.

### 3.4. Extensive Chromosomal Arrangements between Brassica Species

**Figure 5:**
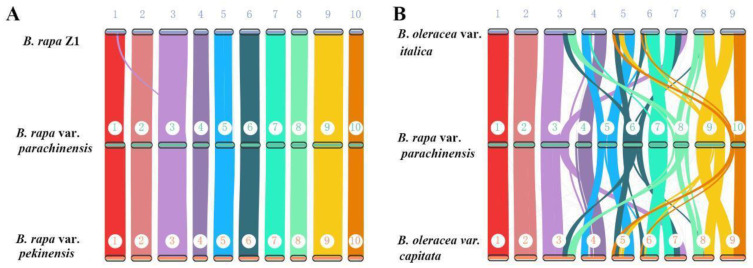
*Genome* synteny based on orthologous genes within and between species for *B. rapa* var. *parachinensis*. (**A**) Genome synteny between *B. rapa* var. *parachinensis* and two other *B. rapa* genome assemblies (*B. rapa* Z1 [11] and *B. rapa* var. *pekinensis* [16]); (**B**) Genome synteny between *B. rapa* var. *parachinensis* and two highly continuous assemblies of the *B. oleracea* genome (*B. oleracea* var. *capitata* [6,11] and *B. oleracea* var. *italica* [11]). Homologous chromosomes are labelled with the same number. “1–10” represent chromosome 1–10, respectively.

Genome-wide synteny analysis was conducted using syntenic orthologous genes both within and between species for *Brassica rapa*. Firstly, the genome of Chinese flowering cabbage was compared to two published genome assemblies of different strains of this species, *B. rapa* Z1 [11] and *B. rapa* var. *pekinensis* [16]. The SyMAP map reveals that these three *Brassica rapa* assemblies retain well conserved overall genome architecture except for a translocation event between chromosome 1 and chromosome 3 that differentiates our assembly to the other two assemblies (Figure 5A). Next, we performed the comparison between *B. rapa* var. *parachinensis* and two highly continuous assemblies of the *B. oleracea* genome [6,11]. Besides the different chromosome numbers (i.e., *B. rapa* var. *parachinensis*; AA genome, *n* = 10 and *B. oleracea*; CC genome, *n* = 9), we observed extensive chromosomal rearrangements between these two species (Figure 5B). Only 2 chromosomes (Chr1 and Chr2) showed minimal changes since their divergence from a common ancestor. The extensive chromosomal rearrangements that occurred during the course of Brassica genome evolution is different from the observation in *Oryza*, one of the well-studied *genus* models in a monocot, in which the karyotype of most diploid species is well-conserved, even over 15 million years evolutionary history [43].

### 3.5. Genome Structure Evolution in Brassica: Insight from Pericentromeric Regions

The pericentromeric regions of plant genomes are among the most rapidly evolving genomic parts, which are found to be largely driven by some major mechanisms such as the proliferation of LTR-retrotransposons, gene conversions, and segmental duplications [44]. Comparison of the pericentromeric regions among three assemblies of the *B. rapa* with different assembly qualities (Supplementary Figure S1E) revealed that the current assembly resolved a larger part of the pericentromeric repetitive regions than the other two assemblies (Supplementary Figure S1A–D). A large part of the pericentromeric regions was missed in the other two assemblies, especially the *B. rapa* var. *pekinensis* assembly. This result shows that high contiguous genome assemblies are required for comparative genomic analysis of highly repetitive regions.

**Figure 6:**
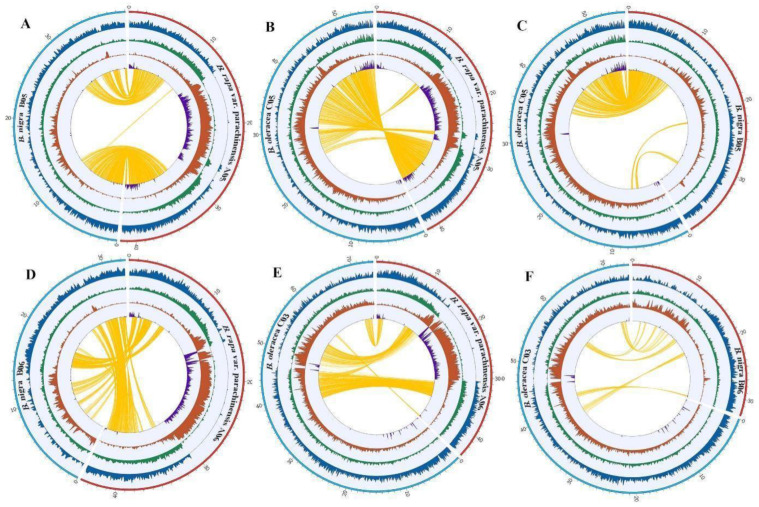
Comparative analysis of sequence features and synteny at the pericentromeric regions on chromosome 5 and 6 among three Brassica genome types: Chinese flowering cabbage (AA genome), *B. nigra* (BB genome) and *B. oleracea* (CC genome). (**A**) Synteny map of Chr05 between *B. nigra* (BB genome) and *B. rapa* var. *parachinensis* (AA genome); (**B**) Synteny map of Chr05 between *B. oleracea* (CC genome) and **B. rapa* var. *parachinensis** (AA genome); (**C**) Synteny map of Chr05 between *B. oleracea* (CC genome) and *B. nigra* (BB genome). (**D**) Synteny map of Chr06 between *B. nigra* (BB genome) and *B. rapa* var. *parachinensis* (AA genome); (**E**) Synteny map of Chr03 of *B. oleracea* (CC genome) and Chr06 of *B. rapa* var. *parachinensis* (AA genome); (**F**) Synteny map of Chr03 of *B. oleracea* (CC genome) and Chr06 of *B. nigra* (BB genome). Tracks in the circos plot from outer to inner represent a: Chromosomes; b: Gene; c: DNA-type TE; d: LTR retrotransposons; e: Tandem repeats; f: Synteny.

Thus, for interspecies comparison, we selected highly contiguous assemblies for two closely related Brassica species, *B. nigra* and *B. oleracea*, which represent two other Brassica genome types (BB and CC) and compared the genome structure and sequence features at the pericentromeric regions of all chromosomes among these three Brassica species or genome types. We found that the pericentromeric regions of chromosome 5 and 6 in *B. rapa* experienced a lineage-specific LTR-retrotransposon amplification history. For example, comparison of chromosome 5 between *B. rapa* and *B. nigra* (Figure 6A) showed that *B. rapa* has a clear enrichment of the LTR retrotransposon compared to the orthologous pericentromeric regions of *B. nigra* although the syntenic relationship of the whole chromosome is well retained between these two species. This difference is more likely to be caused by the lineage specific LTR retrotransposon amplification history since their divergence. While comparison between *B. rapa* and *B. oleracea* (Figure 6B) showed that the synteny of chromosome 5 breaks at the centromere region (see also Figure 5B) and the break event is more likely to occur in the *B. oleracea* lineage since the *B. rapa* shares the synteny block with *B. nigra* (Figure 6A), while the *B. oleracea* does not (Figure 6C). Thus, chromosome rearrangements may be an alternative cause for the different genome structure features observed in the pericentromeric regions. Similarly, the comparison of chromosome 6 revealed an analogous pattern (Figure 6D–F).

### 3.6. Structural Variants in Brassica Genomes

**Figure 7:**
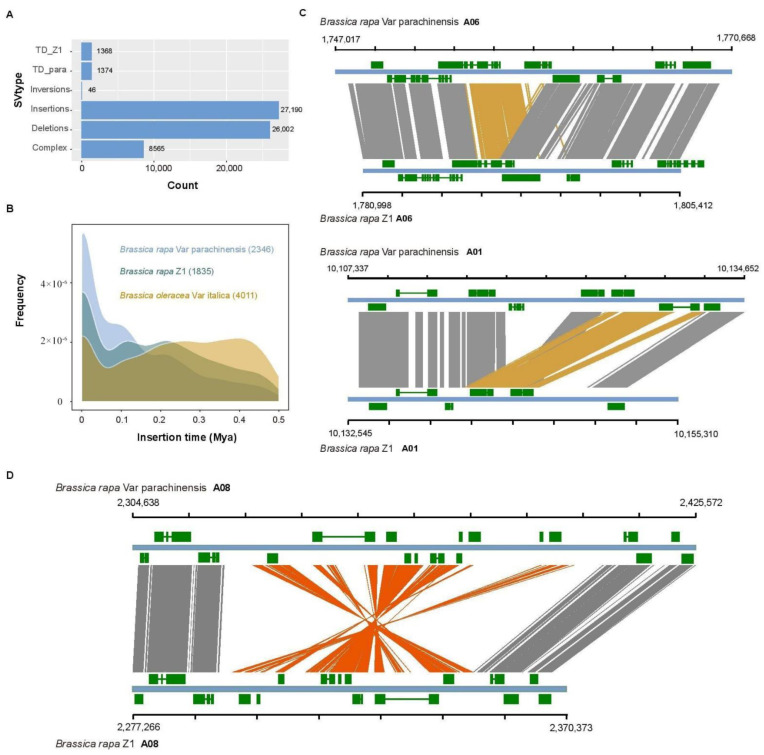
Structural variations between two *B. rapa* lines. (**A**) Total number of structural variations identified using highly continuous assemblies between *Brassica rapa* Z1 and *Brassica rapa* var. *parachinensis*. TD_Z1, tandem duplications in the Z1 assembly relative to the *parachinensis* assembly and TD_pare vice versa. Complex SVs indicate their breakpoints are imprecise. (**B**) Distribution of insertions times of LTR-retrotransposons in three highly continuous Brassica genome assemblies. (**C**) Examples of tandem duplication impacting genes. (**D**) Example of medium size genomic inversions between *Brassica rapa* Z1 and *Brassica rapa* var. **parachinensis*,* which prevails in Brassica genome evolution.

Structural variation (SV) is generally defined as genomic alterations that are 50 bp or larger in size, typically including insertions (INSs), deletions (DELs), duplications (DUPs), inversions (INVs) and translocations (TRAs). SVs greatly impact the genes encoded in the genome and are responsible for diverse agronomically important phenotypes/traits. Compared to single nucleotide polymorphism (SNP) and short insertions and deletions (InDels), SVs are less commonly explored due to the difficulty in fully identifying them with short reads. De novo genome assemblies, especially with high contiguity, can facilitate in-depth genome-wide identification of all forms of structural variations. To the best of our knowledge, no work so far has been conducted to identify SVs based on high-contiguous genome assemblies in Brassica genomes. To close this knowledge gap and have a first glimpse of SVs differing within *Brassica rapa* genomes, we identified SVs using the genomes of *B. rapa* Z1 [11] and *B. rapa* var. *parachinensis* (this study), each with genome assembly contig N50, 5.51 Mb and 7.26 Mb, respectively. As shown in Figure 5A, these two genomes are different only in a single translocation and do not exist in large chromosomal rearrangements. Using the whole genome alignment approach, we identified a total of 27,190 insertions, 26,002 deletions, 1374 duplications in *parachinensis* assembly, 1368 duplications in Z1 *assembly,* and 46 medium-sized inversions with sizes ranging from 5.2 Kb to 1431.6 Kb, and 8565 complex SVs with imprecise breakpoints between Z1 and *parachinensis* (Figure 7A). Of the insertion events, 845 and 847 are found to be newly occurred LTR insertions specifically in *parachinensis* and Z1 assembly, respectively, which are consistent with their relatively recent estimated insertion times (Figure 7B). A large proportion of insertions and deletions detected was found to overlap with the gene regions based on the gene annotation. In Figure 7C, two cases of local tandem duplication are shown to overlap with gene fragments or full genes. Additionally, comparative genomic analysis can also provide insights into the mutational mechanisms of structural variations. Of the 46 inversions identified, we found that repeat sequences, especially inverted repeat sequence features prevail at the flanking regions, highlighting the causal role of sequence features on small-size inversion formation (Figure 7D). Taken together, our analysis of genomic structural variations based on these highly contiguous genome assemblies provide the first glimpse of SVs in the Brassica genomes and their functional significance on gene structure and thus the potential effect on phenotype.

## 4. Discussion

Chinese flowering cabbage (*B. rapa* var. *parachinensis*) is an important leafy and bolting stem vegetable with high nutritional value which has been widely grown in Asia [19]. Among the abundant ecological types of *Brassica rapa* that are planted as vegetables in China, the Chinese flowering cabbage is the one that is well-adapted to the high temperature and high humidity climate in the south of China. It can be planted all year round for tender flower products without the need for a strict vernalization process. In this study, we report a chromosome-level genome assembly of this important ecological *B. rapa* strain, the Chinese flowering cabbage, which provides a valuable genomic data resource for evolutionary studies for *B. rapa* and related Brassica species.

Highly continuous genome assembly is critical for genome-wide marker development and gene model prediction. Enormous studies have demonstrated that recent long-read sequencing technologies can greatly improve the continuity of genome assembly [3,11,13]. In this study, we used PacBio long reads to assemble the *B. rapa* var. *parachinensis* genome. Because of the low heterozygous ratio (0.16%) of the plants used in this genome sequencing, we obtained the contig N50 length of 7.26 Mb, which is longer than the two *B. rapa* genomes sequenced recently by PacBio and Nanopore technology [11,16,17], and much longer than the genomes of *B. rapa* and *B. oleracea* sequenced using Illumina technology [3,6]. We applied the Hi-C technique to scaffold more than 545 Mb contigs onto 10 chromosomes. The N50 length scaffold of the final assembly reached 32.3 Mb, with the maximum size of 47.4 Mb, which was similar to the *B. rapa* Z1 genome sequenced with Nanopore technology [11] (Table S6). The completeness of the genome (97.8%) was validated using the BUSCO analysis in the present study, and surpassed most of the genome of related Brassica species sequenced thus far, including *B. oleracea* HDEM [11], *B. oleracea* var. *botrytis* [12], and *B. rapa* Z1 [11] (Table S6).

In the present study, the assembly of the Chinese flowering cabbage genome resolved most of the pericentromeric regions of the *B. rapa*. Among them, the pericentromeric regions of chromosome 5 (A05) and 6 (A06) were found to be significantly expanded in comparison to other pericentromeric regions and very few genes were annotated in this region (Figure 2B; Figure 6). This observation can further be verified by the Hi-C contact map in which the pericentromeric regions of chromosomes 5 and 6 have a clear sparse Hi-C contact signal that is mostly caused by repetitive sequences (Figure 3). Strikingly, this expansion seems to be lineage specific since we do not observe a similar pattern in the two other Brassica genome types, i.e., chromosome C05 and C06 in *B. oleracea* and *B. napus* [11,13], and chromosome B05 and B06 in *B. nigra* (Figure 6A). This lineage specific expansion may have played a role in the evolutionary divergence of *Brassica* AA, BB, and CC genomes. It is worth noting that such large repetitive regions can only be resolved by long-read sequencing technology. For example, in the previous studies, *B. rapa* Z1 and the *B. napus* AA genome assemblies present a similar but relatively weaker pattern than the current assembly [11,13,16] (Figure S1). However, in the assembly of *B. rapa* [11,13,16] (Figure S1E), sequenced by PacBio Sequel with a N50 of 1.45 Mb, does not present the large repetitive regions in its assembly (Supplementary Figure S1E).

The *genus* Brassica contains three basic genomes, *B. rapa* (AA genome), *B. nigra* (BB genome), and *B. oleracea* (CC genome), which further hybridize to give rise to three allopolyploid species, *B. napus* (AACC genome), *B. juncea* (AABB genome), and *B. carinata* (BBCC genome) [2,12]. In the present study, a phylogenetic tree was constructed to analyze the evolution of the Brassica species. Interestingly, the Chinese flowering cabbage shows the closest relationship with the *B. juncea* AA genome but not with two *B. rapa* genomes (Chinese cabbage and yellow sarson) (Figure 4) [11,16]. The *B. rapa* species can be further subdivided into six populations: turnips (Chinese and European turnips), sarsons (sarson, rapid cycling and spring/winter oilseed), turnip rapes, taicai and mixed Japanese morphotypes, pak choi (pak choi, wutacai, Chinese flowering cabbage, and zicaitai varieties), and heading Chinese cabbages [2]. Our results suggested that the donor of the AA genome in *B. juncea* is most likely from the pak choi group (Chinese flowering cabbage) in contrast to other *B. rapa* varieties, such as sarsons and turnips [11,31]. Meanwhile, we found that *B. rapa* Z1 (sarson) was sister firstly to *B. napus* AA genome and then other AA genomes, implying that it should be the evolutionarily closest donor of the AA genome in *B. napus*. Similarly, the *B. oleracea* can also be subdivided into seven populations such as kohlrabies, Chinese kale, cauliflower, broccoli, Brussels sprouts, kale, and cabbages [2]. Interestingly, *B. oleracea* var. *capitata* (cabbages) was sister firstly to two *B. napus* CC genomes and then with *B. oleracea* var. *italica* (broccoli), implying the donor of the CC genome in *B. napus* probably evolved from *B. oleracea* var. *capitata* (cabbages) (Figure 4). Thus, we demonstrated that high continuity genome assemblies can aid in the interpretation of the evolutionary relationships among Brassica species.

Numerous cases of studies found that structural variations can impact larger genomic regions than SNPs. Structural variant (SV) discovery would not only help our understanding of the landscape of genomic variation within and between species, but also reveal the functional significance of SVs [45]. In comparison to the SVs detection methods that are based on Illumina short reads, the whole assembly-based method can fully recover the SVs in theory but still depend on assembly quality. SVs studies in humans [46,47], and in a wide range of plant species, such as rice [45], Maize [48], tomato [48,49], *Arabidopsis* [49], and *Brassica rapa* [50], indicate that SVs can affect a large proportion of coding genes. In the current study, we detected SVs between the genome assemblies of two *Brassica rapa* lines and identified a total of 27,190 insertions, 26,002 deletions, 1368 duplications, and 46 medium-sized inversions with a size from 5.2 Kb to 1431.6 Kb, and 8565 complex SVs with imprecise breakpoints between them (Figure 7). These SVs may affect coding genes that may further contribute to phenotypic variations, such as morphological and phytochemical characteristics.

In summary, we report a chromosome-level genome assembly of the Chinese flowering cabbage and its accurate gene and TE annotation. The phylogenetic analysis indicates this genome has a closer evolutionary relationship with the AA diploid progenitor of *B. juncea*. We also found the lineage specific pericentromeric expansion events on the chromosomes 5 and 6 of the *Brassica* AA genome compared to the orthologous genomic regions in the *Brassica* BB and CC genomes. Finally, we report a large number of structural variations (SVs) between two *B. rapa* lines (Z1 and *parachinensis*) using high continuity genome assemblies. Overall, our high-quality genome assembly of the Chinese flowering cabbage provides a valuable genetic resource for deciphering the genome evolution of Brassica species and it would serve as the reference genome guiding the molecular breeding practice of *B. rapa* crops.

## Figures and Tables

**Table 1:** Statistics and annotated analysis of the Chinese flowering cabbage genome assembly.

	Number	Size	Sequence Coverage (X)	Percentage (%)
Estimate of genome size		515 Mb		
PacBio reads	4,448,280	113,068 Mb	219.31	
PacBio reads N50		28,414 b		
80X PacBio reads N50		43,902 b		
Illumina reads	322,016,292	42,330 Mb	82.10	
HiC reads	441,545,786	66,231 Mb	128.46	
Total reads		221,630 Mb	429.89	
Contigs	450	384 Mb		74.50
N50 of contigs		7.2 Mb		
Longest contig		19.9 M		
scaffold	69	384 Mb		74.62
N50 of scaffold		32.2 Mb		
Longest scaffold		47.5 Mb		
GC content		144.4 Mb		37.61
Total repetitive sequences		170.3 Mb		44.26
Total protein-coding genes	47,598	47.3 Mb		12.31
Average length per gene		2060 bp		
Average exons per gene	6.13	199 bp		

## Data Availability

The raw genome, RNA sequencing data and Hi-C data were deposited in the China National GeneBank Data Base (CNGBdb) under Bioproject number CNP0001121. The final chromosome assembly was submitted to CNGBdb under the same Bioproject.

## Supplementary Materials

The following supporting information can be downloaded at: https://www.mdpi.com/article/10.3390/plants12132498/s1, Figure S1: Comparative synteny analysis of the pericentromeric regions of Chr05 and Chr06 among three *B. rapa* lines; Table S1. The 20 eudicot species and references; Table S2: Statistics of sample genome characteristics (K-mer = 17); Table S3: Coverage statistics of Chinese flowering cabbage genome using SAM tools; Table S4: Assessment of the completeness of the Chinese flowering cabbage genome assembly by BUSCO (Benchmarking Universal Single-Copy Orthologs); Table S5: Distribution of repeat sequences in Chinese flowering cabbage genome; Table S6: Comparison of the genome assembly between Chinese flowering cabbage (*B. rapa* var. *parachinensis*) and other representative *Brassica* plants.
