# Supplementary material for: A High-Continuity Genome Assembly of Chinese Flowering Cabbage (Brassica rapa var. parachinensis) Provides New Insights into Brassica Genome Structure Evolution
Source: Plants (Basel). 2023 Jun 29;12(13):2498. doi: 10.3390/plants12132498 (PMC10347079; doi:10.3390/plants12132498)
Supplement: Supplementary file 1 [file plants-12-02498-s001.zip › plants-2326680-supplementary.pdf]

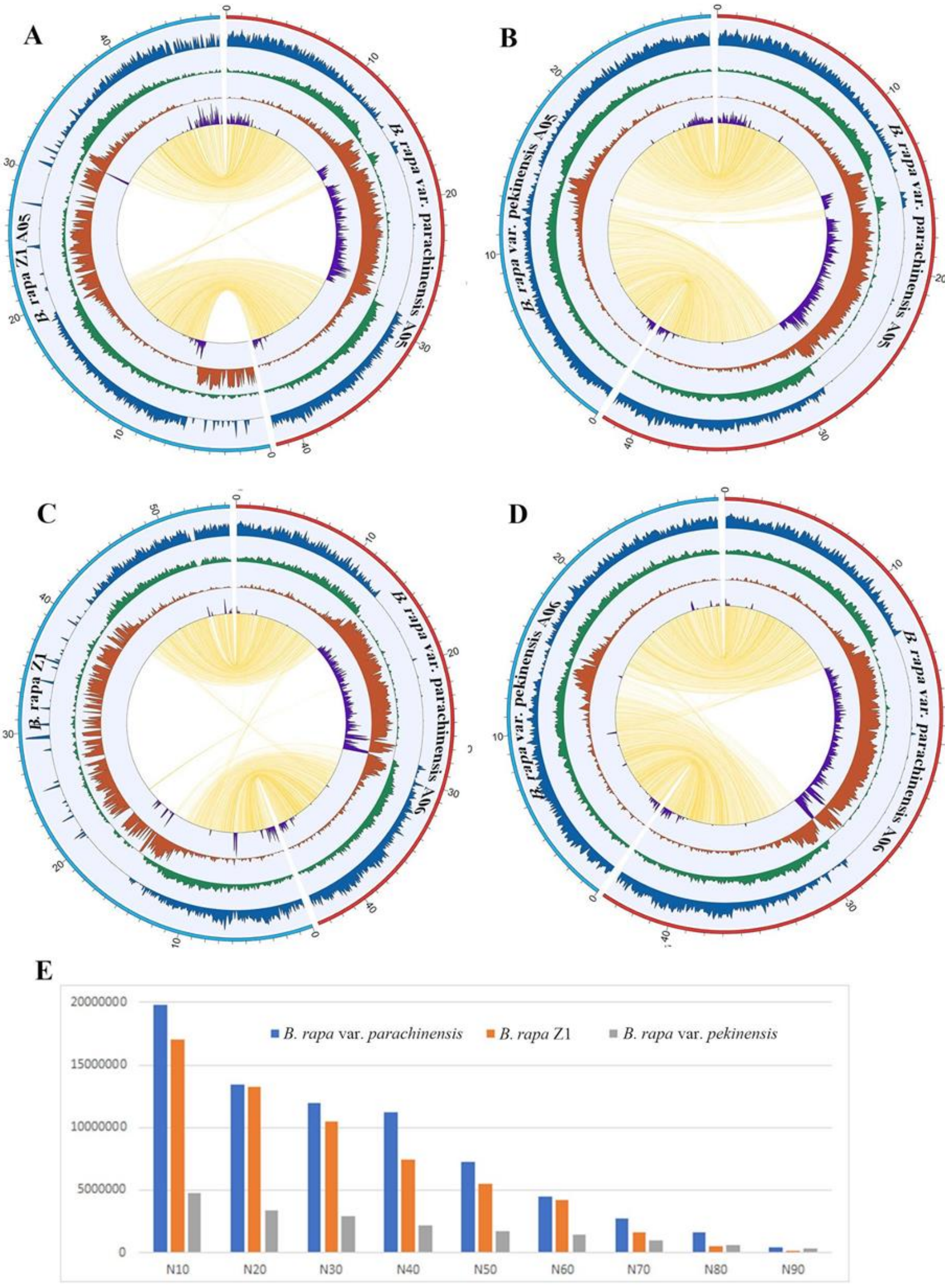

Supplementary Figure S1. Comparative synteny analysis of the pericentromeric regions of Chr05 and Chr06 among three *B. rapa* lines. (A) Synteny map of Chr05 between *B. rapa* Z1 and *B. rapa* var. *parachinensis*; (B) Synteny map of Chr05 between *B. rapa* var. *pekinensis* and *B. rapa* var. *parachinensis*; (C) Synteny map of Chr06 between *B. rapa* Z1 and *B. rapa* var. *parachinensis*. (D) Synteny map of Chr06 between *B. rapa* var. *pekinensis* and *B. rapa* var. *parachinensis*; (E) Summary of contig continuity metrics of the assemblies of three *B. rapa* lines. Tracks in the Circos plot from outer to inner represent: a: Chromosomes; b: Gene; c: DNA-type TE; d: LTR retrotransposons; e: Tandem repeats; f: Synteny.

Table S1 The 20 eudicot species and references

eudicot species	references
Arabidopsis thaliana	The Arabidopsis Information Resource: Making and mining the "gold standard" annotated reference plant genome.
Arabidopsis lyrata	The genome portal of the Department of Energy Joint Genome Institute: 2014 updates.
Capsella rubella	The Capsella rubella genome and the genomic consequences of rapid mating system evolution
Aethionema arabicum	An atlas of over 90,000 conserved noncoding sequences provides insight into crucifer regulatory regions
Thellungiella salsuginea<br>Thellungiella halophila	The Reference Genome of the Halophytic Plant Eutrema salsugineum
Schrenkiella parvula	The genome of the extremophile crucifer Thellungiella parvula
Sisymbrium irio	An atlas of over 90,000 conserved noncoding sequences provides insight into crucifer regulatory regions
Brassica nigra	A highly contiguous genome assembly of Brassica nigra (BB) and revised nomenclature for the pseudochromosomes
Brassica oleracea var italica<br>Brassica rapa Z1	Chromosome-scale assemblies of plant genomes using nanopore long reads and optical maps
Brassica oleracea var capitata	The Brassica oleracea genome reveals the asymmetrical evolution of polyploid genomes
Brassica napus Var shengli3 and Brassica napus Var westar	Eight high-quality genomes reveal pan-genome architecture and ecotype differentiation of Brassica napus
Brassica juncea	Genomic signatures of vegetable and oilseed allopolyploid Brassica juncea and genetic loci controlling the accumulation of glucosinolates
Brassica rapa var pekinesis	Brassica rapa genome 2.0: a reference upgrade through sequence re-assembly and gene re-annotation

Table S2 Statistics of sample genome characteristics（K-mer=17）

Sample	Raw data	K-mer number	K-mer Depth	Genome Size	Heterozygous Ratio (%)	Repeat (%)
Chinese flowering cabbage	47,4Gb	42,330,355,055	79	515.55 Mb	0.16	64.18

Table S3 Coverage statistics of Chinese flowering cabbage genome using SAM tools

		PacBio reads	Illumina reads
Genome	Coverage[a] (%)	98.75%	98.61%
	Coverage at least 4X (%)	98.73%	97.83%
	Coverage at least 10X (%)	98.67%	97.08%
	Coverage at least 20X (%)	98.56%	96.23%
	Average sequencing depth[b]	214.9466	115.6634

[a]Coverage： The proportion of genomes that were covered by reads; [b]Average sequence depth: The average depth of each base on the genome that is covered by reads.

Table S4 Assessment of the completeness of the Chinese flowering cabbage genome assembly by BUSCO (Benchmarking Universal Single-Copy Orthologs)

Species	BUSCO notation assessment results
Chinese flowering cabbage	97.8%[S: 83.9%,D: 13.9%],F: 0.8%,M: 1.4%,n: 1440

BUSCO notation: C: Complete single-copy BUSCOs; S: Complete and single-copy BUSCOs; D: Complete duplicated BUSCOs; F: Fragmented BUSCOs; M: Missing BUSCOs; n: Total BUSCO groups searched.

Table S5 Distribution of repeat sequences in Chinese flowering cabbage genome

Types	Length (Mb)	% in Genome
DNA Transposons	133	17.62
MITE	21	5.47
LTR	103	22.26
Simple repeats	30	7.89
Total	287	53.24

MITE: Miniature inverted transposable elements; LTR: Long terminal repeat.

Table S6 Comparison of the genome assembly between Chinese flowering cabbage (*B. rapa* var. *parachinensis*) and other representative *Brassica* plants

	B. rapa var. *parachinensis*	*B. rapa*. Chiifu	*B. rapa*.Z1	*B. oleracea* HDEM	*B. oleracea* To1000	*B. oleracea* var. botrytis
References	This study	Reference[1]	Reference[2]	Reference[2]	Reference[3]	Reference[4]
Estimated genome size (Mb)	515	442	529	630	630	603
Number of scaffolds	69	1301	335	140	1428	ND
Cumulative size(Mb)	384.70	391.41	401.92	554.97	473.83	ND
N50(Mb) (L50)	32.23 (5)	33.88 (5)	15.38 (8)	29.51 (8)	48.36 (5)	ND
Maximum size(Mb)	47.54	54.54	38.87	48.26	64.98	ND
Number of Ns(Mb)	0.57(0.15%)	ND	32.96(8.20%)	9.95 (1.79%)	42.74 (9.02%)	ND
Number of contigs	450	1498	627	264	51,566	1,484
N50 (Mb)	7.26	1.44	5.51	9.49	0.02	2.11
Maximum size(Mb)	19.90	ND	22.12	26.71	0.16	9.81
Number of genes	47,598	45,985	46,721	61,279	59,225	47,772
BUSCO (complete)	98.6%	97.7%	96.6%	95.8%	95.1%	97.2%
Technology	PacBio and Hi-C	PacBio and Hi-C	Nanopore	Nanopore	Illumina/Roche 454	PacBio

Statistics of the genome assembly between Chinese flowering cabbage (*B. rapa* var. *parachinensis*) and other representative Brassica plants. ND, not determined.

References

1 Zhang L, Cai X, Wu J *et al.* Improved Brassica rapa reference genome by single-molecule sequencing and chromosome conformation capture technologies. *Hortic Res* 2018; **5**: 50.

2 Belser C, Istace B, Denis E *et al.* Chromosome-scale assemblies of plant genomes using nanopore long reads and optical maps. *Nat Plants* 2018; **4**: 879–887.

3 Parkin IAP, Koh C, Tang H *et al.* Transcriptome and methylome profiling reveals relics of genome dominance in the mesopolyploid Brassica oleracea. *Genome Biol* 2014; **15**: R77.

4 Sun D, Wang C, Zhang X *et al.* Draft genome sequence of cauliflower (Brassica oleracea L. var. botrytis) provides new insights into the C genome in Brassica species. *Hortic Res* 2019; **6**: 82.
